# Human exposure to diesel exhaust induces CYP1A1 expression and AhR activation without a coordinated antioxidant response

**DOI:** 10.1186/s12989-023-00559-1

**Published:** 2023-12-08

**Authors:** M. Friberg, A. F. Behndig, J. A. Bosson, Ala Muala, S. Barath, R. Dove, D. Glencross, F. J. Kelly, A. Blomberg, I. S. Mudway, T. Sandström, J. Pourazar

**Affiliations:** 1 Department of Public Health and Clinical Medicine, Umeå University, Umeå, Sweden; 2 Department of Respiratory Medicine and Allergy, Lund University Hospital, Lund, Sweden; 3 Wolfson Institute for Population Health, Barts and The London School of Medicine and Dentistry, Queen Mary University of London, London, UK; 4 MRC Centre for Environment and Health, Imperial College London, London, UK; 5 NIHR Health Protection Research Unit in Environmental Exposures and Health, Imperial College London, London, UK

**Keywords:** Diesel exhaust, Oxidative stress, Xenobiotic metabolism, Aryl hydrocarbon receptor, Immunohistochemistry

## Abstract

**Background:**

Diesel exhaust (DE) induces neutrophilia and lymphocytosis in experimentally exposed humans. These responses occur in parallel to nuclear migration of NF-κB and c-Jun, activation of mitogen activated protein kinases and increased production of inflammatory mediators. There remains uncertainty regarding the impact of DE on endogenous antioxidant and xenobiotic defences, mediated by nuclear factor erythroid 2-related factor 2 (Nrf2) and the aryl hydrocarbon receptor (AhR) respectively, and the extent to which cellular antioxidant adaptations protect against the adverse effects of DE.

**Methods:**

Using immunohistochemistry we investigated the nuclear localization of Nrf2 and AhR in the epithelium of endobronchial mucosal biopsies from healthy subjects six-hours post exposure to DE (PM_10_, 300 µg/m^3^) versus post-filtered air in a randomized double blind study, as a marker of activation. Cytoplasmic expression of cytochrome P450s, family 1, subfamily A, polypeptide 1 (CYP1A1) and subfamily B, Polypeptide 1 (CYP1B1) were examined to confirm AhR activation; with the expression of aldo–keto reductases (AKR1A1, AKR1C1 and AKR1C3), epoxide hydrolase and NAD(P)H dehydrogenase quinone 1 (NQO1) also quantified. Inflammatory and oxidative stress markers were examined to contextualize the responses observed.

**Results:**

DE exposure caused an influx of neutrophils to the bronchial airway surface (p = 0.013), as well as increased bronchial submucosal neutrophil (p < 0.001), lymphocyte (p = 0.007) and mast cell (p = 0.002) numbers. In addition, DE exposure enhanced the nuclear translocation of the AhR and increased the CYP1A1 expression in the bronchial epithelium (p = 0.001 and p = 0.028, respectively). Nuclear translocation of AhR was also increased in the submucosal leukocytes (p < 0.001). Epithelial nuclear AhR expression was negatively associated with bronchial submucosal CD3 numbers post DE (r = −0.706, p = 0.002). In contrast, DE did not increase nuclear translocation of Nrf2 and was associated with decreased NQO1 in bronchial epithelial cells (p = 0.02), without affecting CYP1B1, aldo–keto reductases, or epoxide hydrolase protein expression.

**Conclusion:**

These in vivo human data confirm earlier cell and animal-based observations of the induction of the AhR and CYP1A1 by diesel exhaust. The induction of phase I xenobiotic response occurred in the absence of the induction of antioxidant or phase II xenobiotic defences at the investigated time point 6 h post-exposures. This suggests DE-associated compounds, such as polycyclic aromatic hydrocarbons (PAHs), may induce acute inflammation and alter detoxification enzymes without concomitant protective cellular adaptations in human airways.

**Supplementary Information:**

The online version contains supplementary material available at 10.1186/s12989-023-00559-1.

## Introduction

Traffic-related particulate matter, to which diesel exhaust (DE) is a significant contributor, is associated with increased cardiorespiratory morbidity [1–3]. Many studies have shown that DE exposure triggers acute neutrophilic and lymphocytic inflammation, characterized by the release of a variety of chemokines and cytokines, CXCL8 (IL-8), CXCL1 (Gro-alpha) members of CXC chemokines and Th2 cytokine (IL-13), along with inflammatory cell recruitment to the conducting airways [4–6]. Numerous reports have suggested that the generation of reactive oxidative species (ROS) and the subsequent oxidative stress induced by inhaled particulate matter (PM) and PM associated chemicals, is a key trigger for the observed inflammation [4, 7–9]. This may involve the induction of redox sensitive transcription factors, such as NFkB and c-Jun which belongs to activator protein-1 (AP-1) proteins that can homo or hetero dimer bind to other proteins such as c-fos [10–12]. Little work has focused on the potential mediating role of the induction of cytoprotective antioxidant and xenobiotic enzymes in vivo, such as those under the regulation of the aryl hydrocarbon receptor (AhR) and nuclear factor erythroid 2–related factor 2 (Nrf2).

Diesel exhaust particles (DEPs) characterized by a carbonaceous core with adsorbed organic compounds such as polyaromatic hydrocarbons (PAHs) and quinones. DEP-associated compounds have been suggested to play a major role in inducing oxidative stress and adverse health effects [7]. Previous in vitro studies have demonstrated that DEPs and derived organic extracts cause induction of xenobiotic genes, such as phase I (CYP1A1) and phase II (NQO1) enzymes [13, 14]. Similarly, genes under the regulation of Nrf2, such as heme oxygenase-1, have been shown to be upregulated under low dose DEP challenges of airways cells in vitro, which has been argued to reflect an early adaption to the imposition of oxidative stress [15]. DEP organic compounds have been shown to generate oxidative stress and activate Nrf2 transcription factor [13], which is reported to regulate gene expression of many of human detoxification enzymes that belong to the Antioxidant Response Element (ARE)-gene battery [16, 17].

The role played by the activated AhR is potentially more complex. Beside the activation by exogenous ligands such as poly aromatic hydrocarbons (PAHs), it is noteworthy to mention that endogenous metabolites such as tryptophan and arachidonic acid derivatives have been suggested to be involved in the AhR activation [18, 19]. In this study, we investigated the involvement of detoxification and antioxidant, transcriptions factors and enzymes in the airway defence against DE-induced acute inflammation in healthy volunteers. We hypothesised that inter-individual variation of central airway inflammation would be related to the extent of the activation/nuclear translocation of the Nrf2 and AhR and the induction of cytoprotective enzymes. This was investigated in an exposure study in healthy human subjects exposed to diesel exhaust and filtered air in random blinded order, with bronchoscopy sampling from the airways at 6 h post-exposure, that was based on the protocol from a preceding study by the investigators [4].

## Results

Consistent with previous human challenge studies, DE caused a significant increase in neutrophil numbers (p = 0.013), along with a trend towards an increase in eosinophil numbers in bronchial wash (BW), (p = 0.052, Table 1) [4]. Increased neutrophil, lymphocyte and mast cell numbers were also observed in endobronchial mucosal biopsies 6 h post DE (p < 0.001, p = 0.007 and p = 0.002 respectively, Table 1, Additional file 1: Figure S1). These cell changes occurred in the absence of an increase in Th17 (IL-17A, IL-17F), Th17/Treg (TGFβ) and IL-6 cytokines (Additional file 2: Table S1). The Th1 cytokine, IL-10 was decreased in the BW following DE exposure, (p = 0.019, Additional file 2: Table S1). Though it should be noted that at this early time point, six-hours post-exposure, the measured concentrations were low.

**Table 1:** Inflammation in the bronchial airways

	Air	Diesel	p-value
Bronchial Wash (cells/ml *10^4^)			
Total cells (cells/ml)	6.91 (5.69–9.67)	9.77 (5.52–13.0)	NS
Neutrophils (cells/ml)	1.18 (0.61–1.88)	2.05 (1.02–3.18)	0.013
Macrophages (cells/ml)	5.23 (4.5–8.22)	6.59 (4.13–9.75)	NS
Lymphocytes (cells/ml)	0.24 (0.11–0.38)	0.36 (0.18–0.48)	NS
Eosinophils (cells/ml)	0.00 (0.00–0.01)	0.01 (0.00–0.04)	NS (0.052)
Submucosal cells (cells/mm^2^)			
Neutrophils (cells/mm^2^)	32.30 (17.30–42.90)	72.10 (59.50–82.90)	**< 0.001**
Mast cells (cells/mm^2^)	13.45 (7.53–18.30)	27.20 (18.80–39.10)	**0.002**
CD3^+^ cells (cells/mm^2^)	31.20 (16.30–51.50)	53.60 (26.70–86.90)	**0.007**

Data are presented as median and (interquartile ranges). Cells in the bronchial wash are expressed as cell numbers/ml and submucosal cells are expressed as cell numbers/mm^2^ submucosa area. Comparisons between post air and DE performed using the Wilcoxon-signed-rang-test (n = 16)

All p-values < 0.05 were considered significant, p-values < 0.01 marked bold

The nuclear translocation of p–c-Jun and AhR was increased in the bronchial epithelium after DE exposure (p = 0.004 and p = 0.001 respectively, Table 2, Additional file 1: Figure S1), along with an enhanced CYP1A1 protein expression (p = 0.028, Table 3, Additional file 1: Figure S1). Increased nuclear translocation of AhR was also observed in bronchial submucosal leukocytes after DE exposure (p < 0.001, Table 2, Additional file 1: Figure S1). We saw no evidence of increased nuclear localisation/activation of Nrf2, though a trend to increased total Nrf2 (cytoplasmic + nuclear staining) was seen (2.2-fold, p = 0.06, Table 2, Additional file 1: Figure S1). DE exposure also decreased the NQO1 expression in the bronchial epithelium (p = 0.02, Table 3, Additional file 1: Figure S1) compared to filtered air exposure. While DE exposure increased the CYP1A1 expression, no significant effects on CYP1B1 was observed. There were no changes in the epithelial expression of epoxide hydrolase (EPHX) or Aldo–keto reductases (AKR) following DE exposure (Table 3).

**Table 2:** Transcription factors, total (cytoplasmic + nucleus) and nuclear translocation

	Air	Diesel	p-value
Epi. c-fos total (%)	0.01 (0.00–0.18)	0.02 (0.00–0.11)	NS
Epi. c-fos nucl. (Cell nucleus/mm^2^)	0.00 (0.00–67.70)	30.30 (0.00–121)	NS
Epi. p–c-jun total (%)	0.80 (0.49–1.33)	1.06 (0.79–1.96)	NS
Epi. p–c-jun nucl. (Cell nucleus/mm^2^)	248 (101–345)	362 (227–472)	0.004
Epi. Nrf2 total (%)	1.16 (0.77–2.78)	2.59 (0.61–5.62)	NS (0.06)
Epi. Nrf2 nucl. (Cell nucleus/mm^2^)	270 (148–431)	325 (224–565)	NS
Epi. AhR total (%)	0.38 (0.27–0.76)	0.72 (0.35–1.22)	NS (0.06)
Epi. AhR nucl. (Cell nucleus/mm^2^)	140 (77.50–190)	358 (245–464)	0.001
Subm. leu. AhR nucl. (Cell nucleus/mm^2^)	29.5 (23.2–38.7)	46 (37.2–60.2)	< 0.001

Definition of abbreviations: Epi. = epithelium, nucl. = nucleus, Subm. = submucosal, leu. = leukocyte. Data are presented as median and (interquartile ranges). Total staining (cytoplasmic + nucleus expression), expressed as % of the selected epithelial area. Staining of the nucleus expressed as the number of positively stained nucleus/mm^2^ of the selected epithelial area. Submucosa leukocyte nuclear AhR expressed as the number of positively stained leukocyte nucleus/mm^2^ of the selected submucosa area. Comparisons between post air and DE performed using the Wilcoxon-signed-rang-test (n = 16)

**Table 3:** Epithelial markers of phase 1 and 2 xenobiotic metabolism

	Air	Diesel	p-value
Epi. CYP1A1 (%)	0.31 (0.13–0.55)	0.36 (0.19–1.2)	0.028
Epi. CYP1B1 (%)	0.38 (0.08–0.63)	0.34 (0.17–0.90)	NS
Epi. NQO1 (%)	6.55 (3.96–8.83)	3.42 (2.04–7.08)	0.02
Epi. AKR1A1 (%)	0.59 (0.27–0.87)	0.75 (0.46–0.96)	NS
Epi. AKR1C1 (%)	0.31 (0.10–0.86)	0.61 (0.16–0.98)	NS
Epi. AKR1C3 (%)	1.27 (0.56–2.12)	1.08 (0.45–2.33)	NS
Epi. Epoxide hydrolase (%)	0.020 (0.010–0.036)	0.020 (0.011–0.040)	NS

Definition of abbreviations: Epi. = epithelium. Data are presented as median and (interquartile ranges). Stained epithelial area expressed as % of the selected epithelial area. Comparisons between post air and DE performed using the Wilcoxon-signed-rang-test (n = 16)

**Fig. 1:**
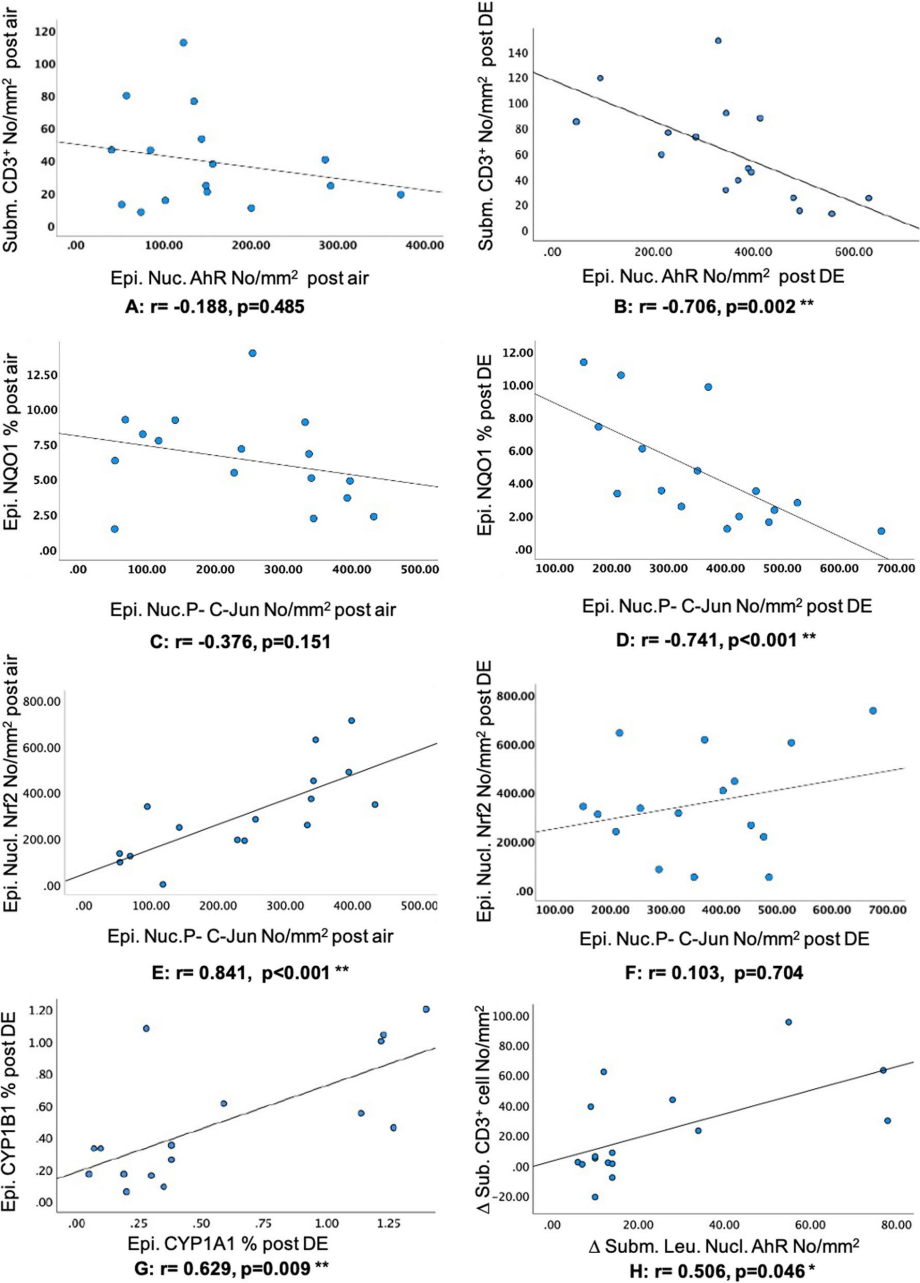
Association of detoxification enzyme, transcription factors and submucosal cells in the endobronchial mucosal biopsies. Definition of abbreviations: Epi. = epithelium, nucl. = nucleus, Subm. = submucosal, leu. = leukocyte. Total staining (cytoplasmic + nucleus expression), expressed as % of the selected epithelial area. Staining of the nucleus expressed as the number of positively stained nuclei/mm^2^ of the selected epithelial area. Submucosal cells and submucosa leukocyte nucleus AhR expressed as the number of positively stained cells and leukocyte nuclei/mm^2^ of the selected submucosa area. Correlation analyses were carried out using Wilcoxon’s nonparametric Spearman’s correlation with a p value 0.05 considered significant. Comparisons in figure A–G, were performed using the absolute values after the air or diesel exhaust exposure while comparisons in figure H, were performed using the change (value post DE exposure minus value post air exposure) in a given parameter, (N = 16). *indicates correlation is significant at the 0.05 level and ** indicates correlation is significant at the 0.01 level

AhR nuclear translocation within bronchial epithelium was negatively associated with the CD3^+^ lymphocyte numbers in the bronchial submucosa. This association was not significant at post air exposure, while DE exposure agumentet the negative association and reached a significant level (r = −0.188, p = 0.485 and r = −0.706, p = 0.002, post air and post DE respectively, (Fig. 1A and B). Epithelial NQO1 expression was negatively associated with epithelial nuclear p–c-Jun expression that did not reach a significant level after air exposure, while after exposure to DE the negative association enhanced to a significant level (r = −0.376, p = 0.151 and r = −0.741, p = 0.001, respectively, Fig. 1C and D). In addition nuclear translocation/activation of Nrf2 was positively associated with nuclear translocation of p–c-Jun at a significant level, post air exposure (r = 0.841, p < 0.001, Fig. 1E). Exposure to DE caused increased c-Jun activity/nuclear translocation in the labsence of increased Nrf2 activity/nuclear translocation and altered the positive association (r = 0.103, p = 0.704, Fig. 1F). Exposure to DE induced significant increase in CYP1A1 expression, while the CYP1B1 expression was not statiscally significant. The positive association between these two CYP variants was relatively robust after DE exposure, (r = 0.629, p = 0.009, Fig. 1G).

The change (expression post DE exposure minus expression post filtered air) in the AhR nuclear translocation within submucosal leukocytes showed a positive association with changes in the submucosal CD^3+^ cells, (r = 0.506, p = 0.046, Fig. 1H). No changes in the concentration of low molecular weight antioxidants (ascorbate, glutathione or urate) were observed in BW 6-h post DE, with no evidence of changes in their oxidation products such as glutathione disulphide and dehydroascorbate (Additional file 3: Table S2).

## Discussion

Consistent with previous observations, DE induced airway neutrophilia and increased number of lymphocytes and mast cells in the endobronchial mucosal biopsies 6-h post-challenge [4]. The proinflammatory effects of DEP have been demonstrated in previous human challenge studies in terms of MAPKs phosphorylation/activation, and increased nuclear localisation of NFkB and AP-1, associated with increased cytokine expression (IL-8, IL-13, Gro-α/CXCL1 [5, 6, 11] as well as EGFR phosphorylation and activation [12]. In the present study, the inflammatory cell responses were shown to be accompanied by a decrease in the IL-10 concentration in the bronchial wash. This may link with our observation in a preceding DE.

study of a skewing towards a Th2 response [6]. Increased nuclear translocation of the transcription factors AhR and phosphorylated c-jun, along with increased protein expression of CYP1A1, indicate the capacity of DE to induce xenobiotic metabolism in the airways, in parallel to the previously reported activation of NFkB and AP-1 [9, 11]. In the current study, the DE challenge caused decreased NQO1 expression without affecting the CYP1B1, EPXH or AKRs enzymes. Similarly to CYP1A1, CYP1B1 and EPXH, AKRs are NAD(P)H-dependent oxidoreductases and are referred to as phase 1 enzymes that play cytoprotective roles in the reduction of a large number of endogenous and/or exogenous carbonyl substrates. Human AKRs genes such as AKR1A1, AKR1C1, AKR1C3 and detoxification enzymes including NQO1 and EPXH genes belong to the ARE-gene battery with their gene expression regulated by Nrf2 activation pathways [16, 17]. In addition activated AhR has been demonstrated to interact directly with ARE-gene battery or indirectly via CYP1A1 and activate Nrf2 to induce NQO1 expression [20]. In the present study the responses such as increased neutrophils, lymphocytes, mast cells and altered detoxification enzyme expression and transcription factors were seen in the absence of activation/nuclear translocation of Nrf2, suggesting the absence of a robust antioxidant and cytoprotective response after an acute high dose diesel challenge. Notably, the total Nrf2 expression (cytoplasic + nuclear) showed a trend to increase, but the activated form of Nrf2 (nuclear translocated), investigated at 6 h post exposure was not changed.

Based on the observations in in vitro cell models, DEPs have been shown to elicit a sequenced activation of redox-sensitive signalling cascades, reflecting changes in the intracellular glutathione (GSH)/glutathione disulphide (GSSG) ratio [15]. This is assumed as an initial adaptation phase, inducing cellular stress though the upregulation of antioxidant and phase I and II xenooobiotic genes. These antioxidant and xenobiotic gene expression are under the regulation of AhR, Nrf2 and AP1. As the oxidative burden increases, NFkB is activated resulting in inflammation and, ultimately, cell death [21]. NQO1 has an important role in the reduction of quinones and functions as a two-electron reductase, minimizing generation of reactive oxygen. NQO1 induction is regulated by the Keap1/Nrf2/ARE pathway to combat oxidative stress. NQO1 can reduce other substrates such as ubiquinone and vitamin E quinone, to form antioxidant protection. A further protective role for NQO1, which is unrelated to its enzymatic activities, is participation in the protein–protein interaction to protect proteasomal degradation such as stabilizing the tumor suppressor p53 [22].

In a previous study, Jaiswal and coworkers suggested that treatment of cells with xenobiotics and antioxidants leads to dissociation of Nrf2 and keap1. The nuclear translocated Nrf2 may then heterodimerize with c-Jun and bind to the ARE, resulting in the induction of NQO1 expression [23]. In this study, in the absence of activated Nrf2, NQO1 expression was decreased within the bronchial epithelium following DE exposure. The epithelial NQO1 expression was inversely associated with expression of the redox-sensitive epithelial nuclear p–c-jun (Fig. 1C, D). Decreased NQO1 expression suggested a DE-induced oxidative stress response which may be related to the lack of activated Nrf2 at the investigated time point. This may represent a time window with an Insufficient antioxidant and cytoprotective response. Phosphorylation of c-jun has been shown to be mediated by MAPKs [24], and a previous human DE exposure study demonstrated activation of redox-sensitive transcription factors and MAPKs [11].

A negative association was seen between epithelial nuclear AhR and submucosal CD3^+^ cells, (Fig. 1A and B). The same negative association trend was seen with submucosal mast cells and epithelial nuclear AhR, but it did not reach a siginificant level either at post air or post DE exposure (p = 0.080 and p = 0.092, respectively). In addition, the positive association between change in the submucosal leukocyte nuclear AhR expression (expression post DE minus expression post air) and change in CD3 counts in the bronchial submucosa (Fig. 1H), was also observed with submucosal mast cells without reaching a significant level (p = 0.076). These associations were not observed for neutrophils investigated in different airway compartments. The in vivo responses observed in the present study suggest that the induction of inflammation preceds an activation of the Nrf2, potentially via the activation of the AhR. What is less clear is the extent to which the activation of AhR contributes toward, or mediates the extent of, individual inflammatory responses.

**Fig. 2:**
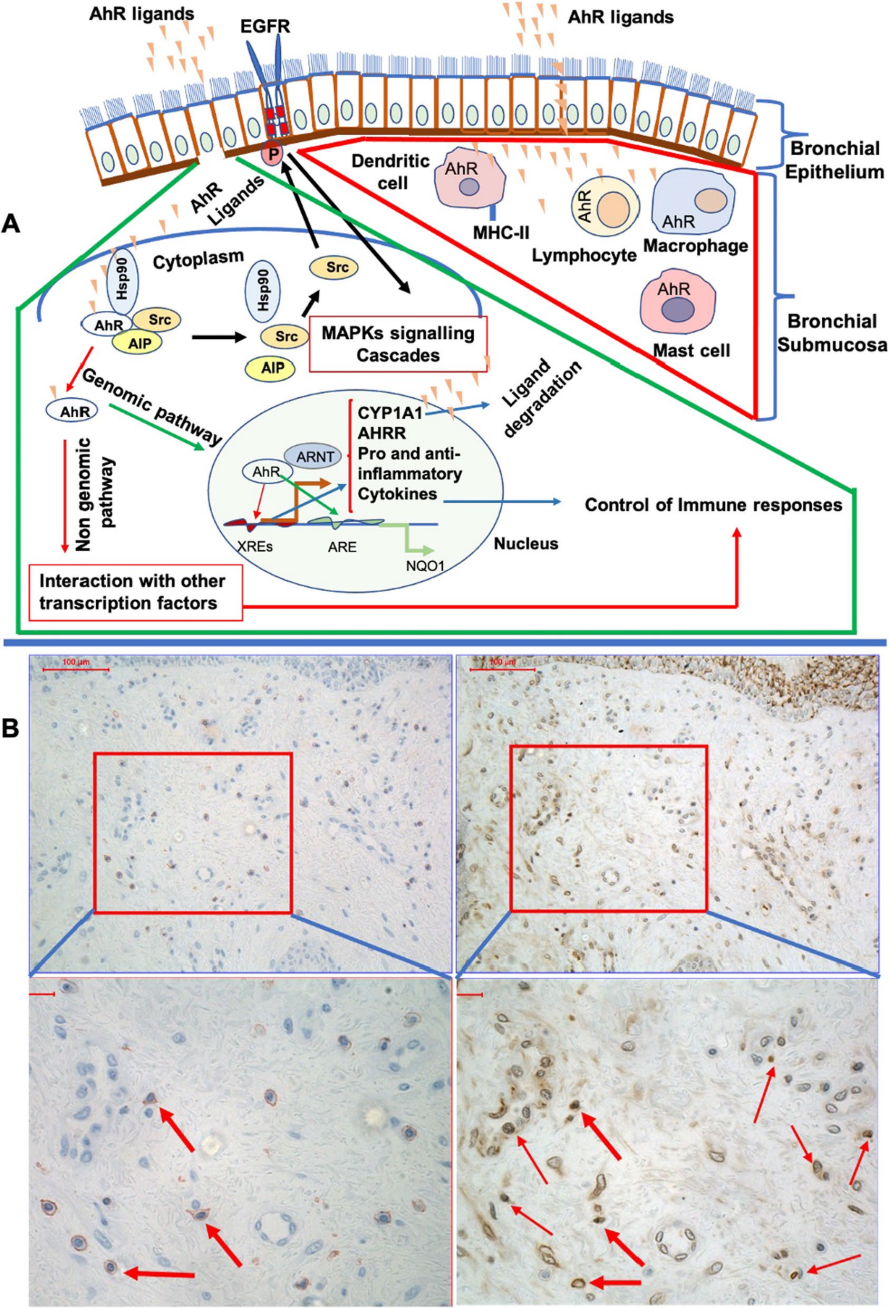
Schematic presentation of AhR activation and co-localization of leukocyte nuclear AhR with CD3 cells. Panel **A** Schematic presentation of AhR activation and interaction with other molecules regulating the induction of genes encoding pro- and anti-inflammatory responses [28, 29]. In the inactive state AhR is found in the cytoplasm associated with protein complexes such as Hsp90 and Src. Following activation, AhR is released from these complexes. The conformationally changed AhR, translocate to the nucleus and dimerizes with Aryl hydrocarbon receptor nuclear translocator (ARNT) factor to target genes such as CYP1A1 and AhRR (aryl hydrocarbon receptor repressor), or to interact with other transcription factors. In addition, the released associated proteins such as Src, may interact with epidermal growth factor receptor (EGFR) and cause MAPKs signalling cascades (Black arrows). The green frame illustrates pathways by which activated AhR, targets genes (genomic pathway) and/or interact with other regulatory molecules (Non genomic pathway) to control these responses in the epithelium. The red frame illustrates AhR within bronchial submucosal leukocytes that may be activated in different cells and involved in the regulation of the immune response. Panel **B** Immunoreactivity within the bronchial submucosa to CD3 antibody (upper left) and corresponding biopsy section stained for AhR antibody (upper right), following DE exposure. Scale bars = 100 μm (micro meters). Lower panel (in panel **B**) images are enlargements of red frames from upper panel (in panel **B**) and show co-localization of CD3^+^ lymphocytes and leukocytes AhR nuclear translocation (bold arrows), narrow arrows show nuclear AhR in leukocytes, Scale bars = 50 μm (micro meters)

The toxicity of PM-associated PAHs is traditionally thought to be mediated by the cytosolic AhR [25–27] a ligand-activated transcription factor complexed with heat-shock protein 90, AhR interacting protein and p23. Following ligand binding, the AhR undergoes nuclear translocation, heterodimerizing with the aryl hydrocarbon nuclear translocator (ARNT) to form the active transcriptional complex, driving the expression of genes containing xenobiotic response elements, including the phase I monooxygenases cytochrome P450 (CYP) 1A1, CYP1A2 and CYP1B1 as well as phase II enzymes, such as NADPH:quinone oxidoreductase (NQO1) (Fig. 2 panel A) [28, 29]. In addition to exogenous ligands, numerous endogenous small molecular weight compounds can also act as AhR ligands, including tryptophan metabolites, indole derivatives and microbiota-derived metabolites [30]. Little work has addressed how these ligands may be modified under air pollution challenges, so it is not clear how the relative balance of exogenous and endogenous ligands contributes to AhR activation post diesel challenge. This will be a critical question to address going forward as transcriptional analyses have shown that: (i) different AhR ligands induce varying responses even within the same cell-type and (ii) the same AhR ligand can induce different responses in different cell-types and tissues [29, 31], which is likely to translate to different functional responses.

In the present study, nuclear translocation of AhR in the epithelium was negatively associated with the number of CD3^+^ cells in the submucosa, (Fig. 1A, B), while the change in the submucosal leukocyte nuclear AhR, was positively associated with the change in submucosal CD3^+^ cell numbers (Fig. 1H). The negative association between epithelia nuclear AhR and submucosal cells such as CD3 cells, may be explained by the role of AhR in inducing the recruitment and differentiation of immune cells such as regulatory T cells. Activated AhR alone or through interaction with other signalling molecules, such as other transcription factors and MAPKs induce recruitment and differentiation of immune cells. In addition, activated AhR can also induce degradation of AhR ligands and consequently downregulation of AhR activation [32]. These different associations within the epithelium and submucosa, could be related to differences in response to DE and/or difference in downstream signalling pathways. The time kinetics of the response and activation of AhR may also be different the in epithelium and submucosal. The present samples were collected and examined at a single time point, six hours post exposure. Further studies are needed to investigate the time kinetics of the responses and activation of AhR.

The increased AhR nuclear translocation in the submucosal leukocytes, may be caused by DE-related AhR ligands, escaping the epithelial barrier and activating AhR in the submucosal leukocytes (Fig. 2 panel B). Diesel exhaust comprise both particulate components and gases, mainly oxides of nitrogen, including NO_2_. We do not suggest NO_2_ to have influenced the epithelial and submucosal inflammatory events by DE. We have carried out studies with NO_2_ at a higher concentration (2 ppm for 4 h) than employed here, without any bronchial mucosal inflammatory responses [33]. Previous studies have shown that fresh DE particles have negligible inherent oxidative potential, compared to ambient PM. For example, determination of oxidative potential (OP) of DEPs (PM 0.1–10) generated under idling engine condition and European Transient Cycle (ETC) urban engine condition was assessed, and demonstrated that oxidizing capacity of DEPs under idling condition was higher than during ETC urban condition. The oxidative properties observed from both these experimental tail-pipe emissions were minor compared to ambient PM [34]. Barath et al. [35] reported that fresh DE particles were abundant in organic species, comparing PAHs from the idling and transient running conditions. The former was shown to have four times higher concentrations of total PAHs in the exposure chamber, mainly related to higher idling gaseous PAH concentrations. PM-associated PAH concentrations were similar under both running conditions, but with differences in profile. PM-associated PAHs including phenanthrene, fluoranthene and pyrene were present in higher concentrations under transient load and speed. However, under idling condition, PM-associated PAHs distribution included heavier PAH (4-rings) species [34]. These PAHs present in the DE are known to be exogenous AhR ligands. In the current study, the epithelial nuclear translocated p–c-Jun expression was positively associated with nuclear translocated/activated Nrf2 at post filtered air exposure, while DE exposure altered this association (Fig. 1E, F). This suggests that higher or lower redox sensitive activity may demand higher/lower Nrf2 activity to keep the antioxidant defense in balance. Decreased NQO1 expression together with absence of Nrf2 activation in this study following DE exposure could potentially be aggravated in more susceptible individuals, such as asthmatic and COPD subjects, with preexisting oxidative stress and/or impaired antioxidant defences [35, 36].

## Conclusion

Six-hours after an acute high-dose diesel challenge, in the presence of a robust central airway neutrophilia and lymphocytosis, we observed evidence of AhR nuclear translocation/activation, associated with increased CYP1A1 expression. This occurred in the absence of Nrf2 activation/nuclear translocation. The present data therefore suggest that the acute effects of diesel exhaust in humans are likely driven by AhR ligands within the organic fraction, activating the AhR. The c-Jun activity as a redox sensitive molecule, in the absence of activated Nrf2 and inflammation, may be secondary to this, as indicated from previous observations in airway nerves [37].

Due to the invasive nature of human studies that employ bronchoscopy, there are limitations when it comes to the number of samples that can be obtained. Therefore, the present study is limited in that it only allows a brief snap-shot in time of multiple overlapping molecular activity. There is clearly a need to obtain a better kinetic understanding of the early induction of these transcription factors and their interaction, but what the present study does imply is that the simple schema, in which inhaled combustion particles first induce oxidative stress in the lung, triggering downstream responses, ranging from adaptation to cell death, does always not seem to be operating and that the AhR, is suggested to play a central role in the early airway response to diesel exhaust. Given our previous observations of the induction of NFκB (p65) and AP-1 (phosphorylated c-jun) at this early time point [11], more work is required to understand how these transcription factors interact with the AhR in the early response, as well as the extent to which the induction of phase I xenobiotic metabolism may result in transient changes in intracellular ROS generation following diesel challenge. However, the role of the AhR in mucosal defences is complex and in interrogating its responses to exogenous ligands, there is also a need to consider whether pathways generating endogenous AhR ligands, such as via induction of indoleamine 2,3-dioxygenase (IDO1), may be playing a role. The evidence presented here suggests that the response of the AhR to exogeous ligands within diesel exhaust is associated with the acute inflammatory response observed.

## Material and methods

### Subjects

Sixteen healthy volunteers (9 females and 7 males), with a mean age of 24 years (range 20–31), were enrolled in the study. All subjects were non-smokers with normal lung function and negative skin prick tests against a standard panel of airborne allergens – see Additional file 4: Table S3. They were all free from respiratory tract infections at least six weeks prior to, and during, the study period. The study was approved by the local Ethical Review Board at Umeå University and performed in accordance with the Declaration of Helsinki, with written informed consent of all participating volunteers.

### DE exposure

All subjects were exposed on two different occasions, once to filtered air and once to diesel engine exhaust, in a randomized order, at least three weeks apart. Each exposure lasted for one hour, during which the subjects alternated between fifteen-minute intervals of rest and exercise on a bicycle ergometer, with the workload adjusted to achieve a minute ventilation of 20 L/min/m^2^ body surface. Diesel exhaust was generated by an idling Volvo diesel engine Volvo (TD45, 4.5 L, 4 Cylinders, 1991, 680 rpm) running on Gasoil E10 (Preem, Sweden). The majority of the exhaust was shunted away, while the remaining part was diluted with filtered air and fed into the exposure chamber where air pollution parameters were continuously monitored. The mean concentration of particulates with a mass median diameter smaller than 10 µm (PM_10_) was 290 ± 27 µg/m^3^ during the diesel exhaust exposures. This was associated with concentrations of nitric oxide (NO) of 2.9 ± 0.37 ppm, nitrogen dioxide (NO_2_) of 0.84 ± 0.10 ppm, total hydrocarbons (HC) of 1.2 ± 0.15 ppm and carbon monoxide (CO) of 2.4 ± 0.53 ppm. Chemical characterization of the exposure emissions has been published elsewhere [34, 38].

### Bronchoscopy and processing of samples

Bronchoscopy was performed six hours after both exposures using a flexible video bronchoscope (Olympus BF IT160, Tokyo, Japan). Endobronchial mucosal biopsies were taken either from the anterior aspect of the main carina and the subcarinae of the 3rd and 4th generation airways of the right side or from the posterior aspect of the main carina and the corresponding subcarinae on the left side. Bronchial wash (BW, 2 × 20 ml) and bronchoalveolar lavage (BAL, 3 × 60 ml) with saline were carried out on the contra-lateral side, in a pre-determined randomized way. The aspirates recovered from the first and second 20 ml instillations of the BW and the pooled BAL were collected into separate siliconized containers placed on ice. All lavage samples were filtered through a nylon filter (pore diameter 100 µm) and centrifuged at 400 g for 15 min. Cell pellets were re-suspended in PBS at a cell concentration of 10^6^ cells/ml. Differential cell counts were performed on slides made by cyto-centrifuge preparation and stained with May-Grünwald Giemsa and 400 cells per slide were counted. Biopsies were fixed overnight (16–20 h) at −20 °C in chilled acetone, containing protease inhibitors (20 mM iodoacetamide and 2 mM phenylmethylsulphonyl fluoride). After fixation the biopsies were processed into glycolmethacrylate (GMA) resins, as described earlier [39].

### Immunohistochemistry

Antibodies for detection of inflammatory cells, including neutrophils and mast cells, were purchased from Dako (Glostrup, Denmark). Human anti CD3, which is a T cell marker, and human anti ECP (EG2), an eosinophil marker, were purchased from Serotec (Oxford, UK) and Diagnostic development (Uppsala, Sweden), respectively. Antibodies used to detect transcription factors and detoxification enzymes, including p–c-Jun, NQO1, c-Fos (mouse monoclonal antibodies), Nrf2 and AHR (rabbit polyclonal antibodies), were purchased from Santa Cruz Biotechnology (Santa Cruz, CA, USA). The supplier for the antibodies against Cyp1A1, Cyp1B1, EPHX (mouse monoclonal antibodies) and Aldo–keto reductases (AKR1A1, AKR1C1 and AKR1C3) was Abcam (Cambridge, UK). Biotinylated rabbit anti-mouse and swine anti-rabbit antibodies were purchased from Dako.

The staining procedure has been previously described in detail [4, 11]. Briefly, the GMA-embedded biopsies were cut in 2-µm thin slices and floated onto ammonia water (1:500). They were collected onto 0.01% poly-L-lysine-coated glass slides and dried at room temperature for one hour. For staining of inflammatory cells, the sections were treated to block endogenous peroxidases and nonspecific antibody binding, and the primary antibody was applied and incubated at room temperature overnight. After rinsing, biotinylated rabbit anti-mouse antibodies against the monoclonal antibodies, and swine anti-rabbit antibodies against polyclonal antibodies (Dako), were applied for two hours, followed by VECTASTAIN Elite ABC kit (Vector Laboratories, Burlingame, USA) for another two hours. Sections for submucosal analysis were developed with aminoethyl carbazole (AEC) as substrate. All sections were counterstained with Mayer´s hematoxylin. Sections where the primary antibody was omitted served as negative controls. The staining procedure was partially modified for transcription factors and detoxification enzymes. Supplementary steps were added in order to increase the permeability of the cells. Sections for epithelial measurements were developed as a brown colour with 3,3-diaminobenzidine (DAB). A detailed description of the staining procedure has previously been published [11].

**Fig. 3:**
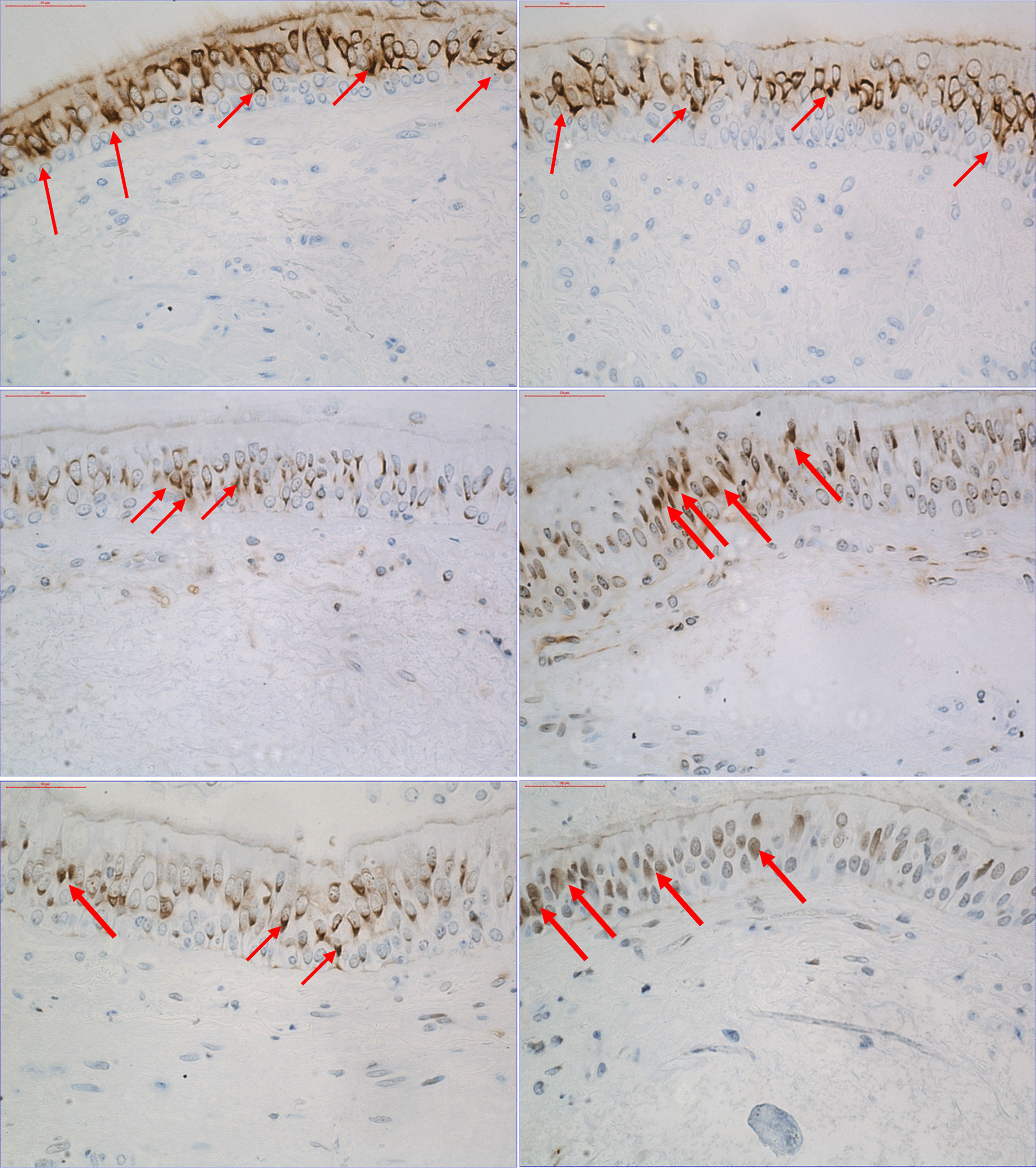
Immunoreactivity within the bronchial epithelium. Upper left shows immunoreactivity to NQO1 post-air exposure and upper right shows NQO1 post-DE exposure. Middle panel shows immunoreactivity to AhR antibody, middle left, post-air exposure and middle right, post-DE exposure. Immunoreactivity to P–C-Jun antibody is shown in lower panel, with lower left at post-air exposure and lower right at post-DE exposure. Narrow arrows indicate positive intracytoplasmic staining and bold arrows indicate nuclear staining. Scale bars = 50 μm (micro meters)

Quantification of the immunohistochemical staining and other analyses were performed by one person who was unaware of how the coded samples corresponding to the exposures. All analyses were done double blinded, with codes broken only after completion of the full analysis and statistics. When quantifying the immunohistochemical staining, a light microscope was used to count inflammatory cells according to their immunoreactivity with specific antibodies in the epithelium and the submucosa respectively, excluding mucosal glands, blood vessels and smooth muscle. A computer-assisted image analysis program (Leica Q500IW, Leica Cambridge UK) was used to calculate the length of the epithelium and the area of the submucosa. Cell counts were expressed as cells/mm^2^ in the selected submucosa area. The images presented in (Fig. 3), displays the immunoreactivity patterns for transcription factors and enzymes in the bronchial epithelium. Quantification of transcription factors in the bronchial epithelium, total staining (cytoplasmic plus nuclear) and enzymes, immunoreactivity was quantified using a Leica DFC 320 camera (Cambridge, UK). The camera was connected to a Leica imaging workstation, specific PC software (Leica Q500IW; Leica, Cambridge, UK). Detection of an appropriate color was quantified using binary definition of color images as displayed on the screen. The binary image required the user to define which pixel in the image was to be considered for measurement. The total staining (cytoplasmic plus nuclear) and enzymes were expressed as the percentage of the total epithelial area showing positive immunoreactivity to the antibodies. It was possible to distinguish between nuclear and cytoplasmic staining using a light microscope. Positive staining of the nucleus was expressed as the number of positive nuclei/mm^2^ of selected epithelium area.

Due to lack of double staining or sequential sections for co-localization, we were not able to quantify nuclear translocated AhR in specific cells in the submucosa. Therefore, AhR activation in the submucosal leukocytes was expressed as the number of nuclear translocated AhR in the leukocytes/mm^2^ submucosa area. Despite to the lack of double staining or sequential sections for co-localization, we could confirm, AhR leukocyte nuclear translocation and co-localization with submucosal CD3^+^ cells post DE exposure ( Fig. 2 panel B).

### Antioxidant analysis

Cell-free BW and BAL supernatants were analysed for total protein, GSH, GSSG, vitamin C (AA and DHA), and UA concentration, as previously described [40]. Briefly, total glutathione concentrations were measured using the GSSG-reductase-DTNB recycling method. AA and UA were measured simultaneously by reverse phase HPLC with electrochemical detection, as previously described [40]. Total vitamin C (DHA + AA) was measured by pre-treating samples with 50 mM Tris(2-carboxylethyl) phosphine for 15 min to reduce DHA and then performing the lipid extraction and HPLC analysis as described above. The DHA concentration was then calculated by subtracting the AA concentration from the total vitamin C concentration.

### Cytokine analyses

Cytokine concentrations were determined in untreated lavage fluids.

IL-17A, IL-17F and TGF-β1 concentrations were measured with commercial ELISA kits (Gen-Probe Diaclone, France) and IL-17E concentrations was determined by using Human IL-17E ELISA Construction Kit (Antigenix America). IL-6 and IL-10 concentrations were measured using high-sensitivity ELISA kits, R&D Systems (Abingdon United Kingdom), which the lower limit detection for IL-6 was 0.007–0.090 (mean 0.031) and for IL-10 was 0.03–0.17 (mean 0.09) for IL-10. Here a lower limit of detection for both IL-6 and IL-10 have been determined by adding 3SD of O.D. blank value. The calculated concentrations below the lower standard point, but above the sensitity to detect minimum dose, recommended in the kit, were accepted as valid values.

### Statistical analysis

Subjects acted as their own controls, and comparison of post-air exposure and post-diesel exhaust exposure were performed using Wilcoxon’s nonparametric signed-rank test. A p value of 0.05, or less, was considered significant and data are presented as median and interquartile range. Correlation analyses were carried out using Spearman’s rank order correlation with a p value of 0.05, or less, was considered significant. Comparisons were performed using absolute value in a given parameter after air or diesel exposure. Comparisons of the change in a given parameter were performed using (post-diesel value minus post-air value). All statistical analyses were performed using SPSS version 27.0 (SPSS Inc., Chicago, USA). Graphical presentation of absolute value in a given parameter after air and diesel for each subject were performed using GraphPad software, prism version 9 (San Diego, CA, USA).

### Supplementary Information

**Additional file 1: Fig. S1.** Transcription factors, enzymes and submucosal cell expression, graphically presentation of air and DE data points. Definition of abbreviations: Epi. = epithelium, nucl. = nucleus, Subm. = submucosal, leu. = leukocyte. Each data point (absolute value post-air and post-deisel for each subject) given graphically, performed using GraphPad software, prism version 9. Total staining (cytoplasmic + nucleus expression) and enzyme staining, expressed as % of the selected epithelial area. Staining of the nucleus expressed as the number of positively stained nuclei/mm2 of the selected epithelial area. Submucosal leukocyte nuclear AhR and submucosal cells are expressed as nuclei or cell numbers/mm2 submucosa area, (n = 16).

**Additional file 2. Table S1. **BW cytokine expression.

**Additional file 3. Table S2. **Bronchial wash antioxidant concentrations.

**Additional file 4. Table S3. **Subject demographics.

## Data Availability

All relevant data are included in the manuscript and supporting information. These are also available from the authors upon reasonable request.

## References

[1] Gauderman WJ, Avol E, Gilliland F, Vora H, Thomas D, Berhane K (2004). The effect of air pollution on lung development from 10 to 18 years of age. N Engl J Med.

[2] Russell AG, Brunekreef B (2009). A focus on particulate matter and health. Environ Sci Technol.

[3] Mills NL, Donaldson K, Hadoke PW, Boon NA, MacNee W, Cassee FR (2009). Adverse cardiovascular effects of air pollution. Nat Clin Pract Cardiovasc Med.

[4] Salvi S, Blomberg A, Rudell B, Kelly F, Sandstrom T, Holgate ST (1999). Acute inflammatory responses in the airways and peripheral blood after short-term exposure to diesel exhaust in healthy human volunteers. Am J Respir Crit Care Med.

[5] Salvi SS, Nordenhall C, Blomberg A, Rudell B, Pourazar J, Kelly FJ (2000). Acute exposure to diesel exhaust increases IL-8 and GRO-alpha production in healthy human airways. Am J Respir Crit Care Med.

[6] Pourazar J, Frew AJ, Blomberg A, Helleday R, Kelly FJ, Wilson S (2004). Diesel exhaust exposure enhances the expression of IL-13 in the bronchial epithelium of healthy subjects. Respir Med.

[7] Xia T, Korge P, Weiss JN, Li N, Venkatesen MI, Sioutas C (2004). Quinones and aromatic chemical compounds in particulate matter induce mitochondrial dysfunction: implications for ultrafine particle toxicity. Environ Health Perspect.

[8] Behndig AF, Mudway IS, Brown JL, Stenfors N, Helleday R, Duggan ST (2006). Airway antioxidant and inflammatory responses to diesel exhaust exposure in healthy humans. Eur Respir J.

[9] Mudway IS, Stenfors N, Duggan ST, Roxborough H, Zielinski H, Marklund SL (2004). An in vitro and in vivo investigation of the effects of diesel exhaust on human airway lining fluid antioxidants. Arch Biochem Biophys.

[10] Kelly FJ (2003). Oxidative stress: its role in air pollution and adverse health effects. Occup Environ Med.

[11] Pourazar J, Mudway IS, Samet JM, Helleday R, Blomberg A, Wilson SJ (2005). Diesel exhaust activates redox-sensitive transcription factors and kinases in human airways. Am J Physiol Lung Cell Mol Physiol.

[12] Pourazar J, Blomberg A, Kelly FJ, Davies DE, Wilson SJ, Holgate ST (2008). Diesel exhaust increases EGFR and phosphorylated C-terminal Tyr 1173 in the bronchial epithelium. Part Fibre Toxicol.

[13] Baulig A, Garlatti M, Bonvallot V, Marchand A, Barouki R, Marano F (2003). Involvement of reactive oxygen species in the metabolic pathways triggered by diesel exhaust particles in human airway epithelial cells. Am J Physiol Lung Cell Mol Physiol.

[14] Bonvallot V, Baeza-Squiban A, Baulig A, Brulant S, Boland S, Muzeau F (2001). Organic compounds from diesel exhaust particles elicit a proinflammatory response in human airway epithelial cells and induce cytochrome p450 1A1 expression. Am J Respir Cell Mol Biol.

[15] Xiao GG, Wang M, Li N, Loo JA, Nel AE (2003). Use of proteomics to demonstrate a hierarchical oxidative stress response to diesel exhaust particle chemicals in a macrophage cell line. J Biol Chem.

[16] Penning TM (2017). Aldo-keto reductase regulation by the Nrf2 system: implications for stress response, chemotherapy drug resistance, and carcinogenesis. Chem Res Toxicol.

[17] Walsh J, Jenkins RE, Wong M, Olayanju A, Powell H, Copple I (2014). Identification and quantification of the basal and inducible Nrf2-dependent proteomes in mouse liver: biochemical, pharmacological and toxicological implications. J Proteomics.

[18] Nguyen LP, Bradfield CA (2008). The search for endogenous activators of the aryl hydrocarbon receptor. Chem Res Toxicol.

[19] Esser C, Rannug A, Stockinger B (2009). The aryl hydrocarbon receptor in immunity. Trends Immunol.

[20] Kohle C, Bock KW (2007). Coordinate regulation of Phase I and II xenobiotic metabolisms by the Ah receptor and Nrf2. Biochem Pharmacol.

[21] Kumar A, Dailey LA, Swedrowska M, Siow R, Mann GE, Vizcay-Barrena G (2016). Quantifying the magnitude of the oxygen artefact inherent in culturing airway cells under atmospheric oxygen versus physiological levels. FEBS Lett.

[22] Dinkova-Kostova AT, Talalay P (2010). NAD(P)H:quinone acceptor oxidoreductase 1 (NQO1), a multifunctional antioxidant enzyme and exceptionally versatile cytoprotector. Arch Biochem Biophys.

[23] Jaiswal AK (2000). Regulation of genes encoding NAD(P)H:quinone oxidoreductases. Free Radic Biol Med.

[24] Pulverer BJ, Kyriakis JM, Avruch J, Nikolakaki E, Woodgett JR (1991). Phosphorylation of c-jun mediated by MAP kinases. Nature.

[25] Soontjens C, Holmberg K, Westerholm R, Rafter J (1997). Characterisation of polycyclic aromatic compounds in diesel exhaust particulate extract responsible for aryl hydrocarbon receptor activity. Atmos Environ.

[26] Misaki K, Suzuki M, Nakamura M, Handa H, Iida M, Kato T (2008). Aryl hydrocarbon receptor and estrogen receptor ligand activity of organic extracts from road dust and diesel exhaust particulates. Arch Environ Contam Toxicol.

[27] Totlandsdal AI, Lag M, Lilleaas E, Cassee F, Schwarze P (2015). Differential proinflammatory responses induced by diesel exhaust particles with contrasting PAH and metal content. Environ Toxicol.

[28] Hao N, Whitelaw ML (2013). The emerging roles of AhR in physiology and immunity. Biochem Pharmacol.

[29] Poulain-Godefroy O, Boute M, Carrard J, Alvarez-Simon D, Tsicopoulos A, de Nadai P (2020). The aryl hydrocarbon receptor in asthma: friend or foe?. Int J Mol Sci.

[30] Rothhammer V, Quintana FJ (2019). The aryl hydrocarbon receptor: an environmental sensor integrating immune responses in health and disease. Nat Rev Immunol.

[31] Denison MS, Faber SC (2017). And now for something completely different: diversity in ligand-dependent activation of Ah receptor responses. Curr Opin Toxicol.

[32] Gutierrez-Vazquez C, Quintana FJ (2018). Regulation of the immune response by the aryl hydrocarbon receptor. Immunity.

[33] Blomberg A, Krishna MT, Bocchino V, Biscione GL, Shute JK, Kelly FJ (1997). The inflammatory effects of 2 ppm NO_2_ on the airways of healthy subjects. Am J Respir Crit Care Med.

[34] Barath S, Mills NL, Lundback M, Tornqvist H, Lucking AJ, Langrish JP (2010). Impaired vascular function after exposure to diesel exhaust generated at urban transient running conditions. Part Fibre Toxicol.

[35] Kelly FJ, Mudway I, Blomberg A, Frew A, Sandstrom T (1999). Altered lung antioxidant status in patients with mild asthma. Lancet.

[36] Barnes PJ (2020). Oxidative stress-based therapeutics in COPD. Redox Biol.

[37] Robinson RK, Birrell MA, Adcock JJ, Wortley MA, Dubuis ED, Chen S (2018). Mechanistic link between diesel exhaust particles and respiratory reflexes. J Allergy Clin Immunol.

[38] Lucking AJ, Lundback M, Mills NL, Faratian D, Barath SL, Pourazar J (2008). Diesel exhaust inhalation increases thrombus formation in man. Eur Heart J.

[39] Britten KM, Howarth PH, Roche WR (1993). Immunohistochemistry on resin sections: a comparison of resin embedding techniques for small mucosal biopsies. Biotech Histochem.

[40] Dove RE, Leong-Smith P, Roos-Engstrand E, Pourazar J, Shah M, Behndig AF (2015). Cigarette smoke-induced induction of antioxidant enzyme activities in airway leukocytes is absent in active smokers with COPD. Eur Clin Respir J..

