# Supplementary figures and images for: Human exposure to diesel exhaust induces CYP1A1 expression and AhR activation without a coordinated antioxidant response
Source: Part Fibre Toxicol. 2023 Dec 8;20:47. doi: 10.1186/s12989-023-00559-1 (PMC10704793; doi:10.1186/s12989-023-00559-1)

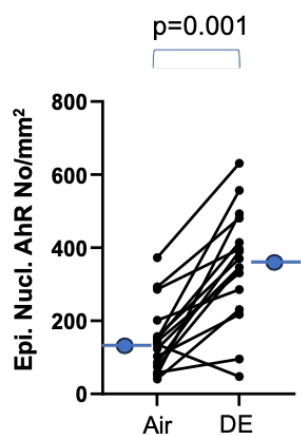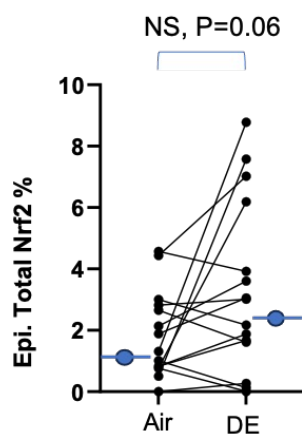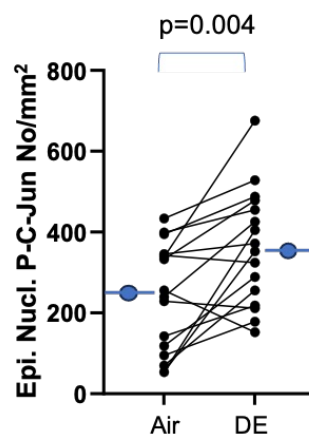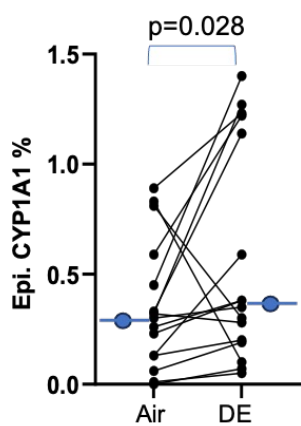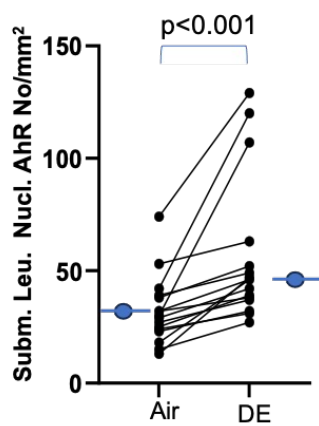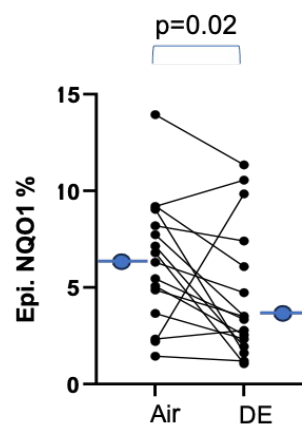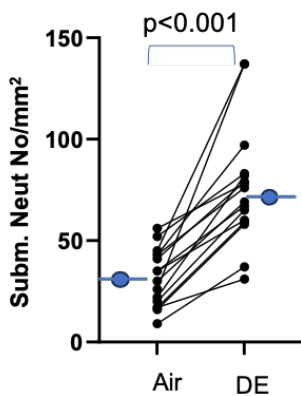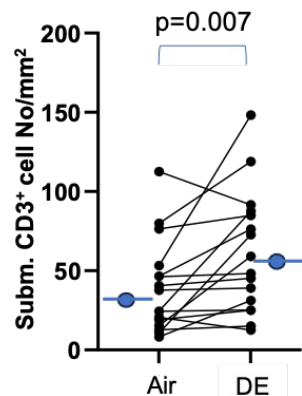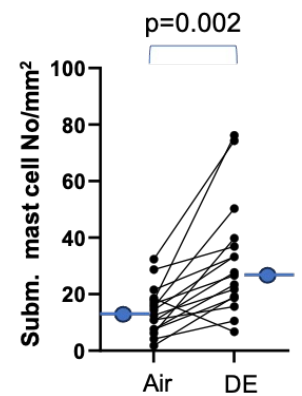

● = median

Supplement: Supplementary file 1 — Additional file 1: Fig. S1. Transcription factors, enzymes and submucosal cell expression, graphically presentation of air and DE data points. Definition of abbreviations: Epi. = epithelium, nucl. = nucleus, Subm. = submucosal, leu. = leukocyte. Each data point (absolute value post-air and post-deisel for each subject) given graphically, performed using GraphPad software, prism version 9. Total staining (cytoplasmic + nucleus expression) and enzyme staining, expressed as % of the selected epithelial area. Staining of the nucleus expressed as the number of positively stained nuclei/mm2 of the selected epithelial area. Submucosal leukocyte nuclear AhR and submucosal cells are expressed as nuclei or cell numbers/mm2 submucosa area, (n = 16). [file 12989_2023_559_MOESM1_ESM.pdf]
