# Supplementary material for: Human exposure to diesel exhaust induces CYP1A1 expression and AhR activation without a coordinated antioxidant response
Source: Part Fibre Toxicol. 2023 Dec 8;20:47. doi: 10.1186/s12989-023-00559-1 (PMC10704793; doi:10.1186/s12989-023-00559-1)
Supplement: Supplementary file 2 — Additional file 2. Table S1. BW cytokine expression. [file 12989_2023_559_MOESM2_ESM.docx]

**Table S1: BW cytokine expression**

|  | **Air** | **Diesel** | **p-value** |
| --- | --- | --- | --- |
| IL-6 (pg/ml) | 4.0 (3.3-7.0) | 4.0 (2.7-11.1) | NS |
| IL-10 (pg/ml) | 0.8 (0.4-1.1) | 0.4 (0.4-0.6) | 0.019 |
| IL-17A (pg/ml) | 1.7 (0.9-2.3) | 1.5 (0.9-2.1) | NS |
| IL17F (pg/ml) | 5.4 (3.5-6.8) | 4.4 (2.9-7.0) | NS |
| TGF-β1 (pg/ml) | 10.6 (8.4-17.9) | 14.3 (8.7-25.1) | NS |

Data are presented as median and (interquartile ranges). Comparisons between post air and DE performed using the Wilcoxon-signed-rang-test (n=16).
