# Supplementary material for: Human exposure to diesel exhaust induces CYP1A1 expression and AhR activation without a coordinated antioxidant response
Source: Part Fibre Toxicol. 2023 Dec 8;20:47. doi: 10.1186/s12989-023-00559-1 (PMC10704793; doi:10.1186/s12989-023-00559-1)
Supplement: Supplementary file 3 — Additional file 3. Table S2. Bronchial wash antioxidant concentrations. [file 12989_2023_559_MOESM3_ESM.docx]

**Table S2: Bronchial wash antioxidant concentrations**

|  | **Air** | **Diesel** | **p-value** |
| --- | --- | --- | --- |
| Total GSH (µM) | 1.39 (1.20-1.61) | 1.21 (0.86-1.66) | NS |
| GSH (µM) | 0.67 (0.43-0.93) | 0.56 (0.29-0.99) | NS |
| GSSG (µM) | 0.38 (0.27-0.45) | 0.31 (0.22-0.45) | NS |
| % GSSG | 25.5 (18.5-29.8) | 29.3 (18.2-35.4) | NS |
| Total vitamin C (µM) | 0.43 (0.40-0.56) | 0.43 (0.40-0.52) | NS |
| Ascorbic acid (µM | 0.35 (0.00-0.46) | 0.00 (0.00-0.51) | NS |
| DHA (µM) | 0.13 (0.08-0.38) | 0.37 (0.08-0.42) | NS |
| % DHA | 18.0 (13.8-21.7) | 17.1 (4.8-18.5) | NS |
| Urate (µM) | 0.37 (0.24-0.42) | 0.27 (0.22-0.40) | NS |

Data are presented as median and (interquartile ranges). Comparisons between post air and DE performed using the Wilcoxon-signed-rang-test (n=16).
