# Supplementary material for: Human exposure to diesel exhaust induces CYP1A1 expression and AhR activation without a coordinated antioxidant response
Source: Part Fibre Toxicol. 2023 Dec 8;20:47. doi: 10.1186/s12989-023-00559-1 (PMC10704793; doi:10.1186/s12989-023-00559-1)
Supplement: Supplementary file 4 — Additional file 4. Table S3. Subject demographics. [file 12989_2023_559_MOESM4_ESM.docx]

**Table S3: Subject demographics**

| **Subject** | **Gender** | **Age (Yrs)** | **FVC (L)** | **FEV_1_ (L)** | **FEV_1_ predicted %** |
| --- | --- | --- | --- | --- | --- |
| 1 | Male | 24 | 4.45 | 4.25 | 94 |
| 2 | Male | 31 | 7.30 | 6.09 | 116 |
| 3 | Male | 27 | 4.53 | 3.70 | 91 |
| 4 | Female | 22 | 4.02 | 3.53 | 89 |
| 5 | Male | 25 | 4.80 | 4.15 | 98 |
| 6 | Male | 28 | 6.60 | 5.30 | 108 |
| 7 | Male | 26 | 5.39 | 4.62 | 97 |
| 8 | Female | 22 | 3.95 | 3.60 | 106 |
| 9 | Female | 22 | 4.04 | 3.55 | 92 |
| 10 | Female | 22 | 4.45 | 4.30 | 113 |
| 11 | Male | 20 | 5.05 | 4.00 | 81 |
| 12 | Female | 25 | 4.75 | 4.05 | 114 |
| 13 | Female | 23 | 3.90 | 3.63 | 108 |
| 14 | Female | 22 | 5.35 | 4.55 | 122 |
| 15 | Female | 25 | 5.70 | 5.15 | 128 |
| 16 | Female | 22 | 4.03 | 3.58 | 96 |
| Mean | 7 Male | 24 | 4.89 | 4.25 | 103.3 |
| SD | 9 Female | 2.9 | 0.98 | 0.74 | 13.1 |
